# Automated Identification of Dementia Using FDG-PET Imaging

**DOI:** 10.1155/2014/421743

**Published:** 2014-02-02

**Authors:** Yong Xia, Shen Lu, Lingfeng Wen, Stefan Eberl, Michael Fulham, David Dagan Feng

**Affiliations:** ^1^Shaanxi Provincial Key Lab of Speech & Image Information Processing (SAIIP), School of Computer Science, Northwestern Polytechnical University, Xi'an 710072, China; ^2^Biomedical and Multimedia Information Technology (BMIT) Research Group, School of Information Technologies, The University of Sydney, Sydney, NSW 2006, Australia; ^3^Department of Molecular Imaging, Royal Prince Alfred Hospital, Sydney, NSW 2050, Australia; ^4^Sydney Medical School, The University of Sydney, Sydney, NSW 2006, Australia; ^5^Med-X Research Institute, Shanghai Jiao Tong University, Shanghai 200030, China

## Abstract

Parametric FDG-PET images offer the potential for automated identification of the different dementia syndromes. However, various existing image features and classifiers have their limitations in characterizing and differentiating the patterns of this disease. We reported a hybrid feature extraction, selection, and classification approach, namely, the GA-MKL algorithm, for separating patients with suspected Alzheimer's disease and frontotemporal dementia from normal controls. In this approach, we extracted three groups of features to describe the average level, spatial variation, and asymmetry of glucose metabolic rates in 116 cortical volumes. An optimal combination of features, that is, capable of classifying dementia cases was identified by a genetic algorithm- (GA-) based method. The condition of each FDG-PET study was predicted by applying the selected features to a multikernel learning (MKL) machine, in which the weighting parameter of each kernel function can be automatically estimated. We compared our approach to two state-of-the-art dementia identification algorithms on a set of 129 clinical cases and improved the performance in separating the dementia types, achieving accuracy of 94.62%. There is a very good agreement between the proposed automated technique and the diagnosis made by clinicians.

## 1. Introduction

Dementia is a chronic and progressive brain disorder, that is, characterized by the progressive loss of memory and cognitive impairment with an attendant disruption of normal daily activities [1]. In 2010, it was estimated that 35.6 million people worldwide were suffering from dementia and it is predicted that this number will double every 20 years [2]. Dementia is now a global health and social problem [3]. The common types of dementia include Alzheimer's disease (AD), vascular dementia (VD), Lewy body dementia (LBD), and frontotemporal dementia (FTD). AD accounts for about 65% of the cases [4]. FTD is the second most common and accounts for between 4% and 20% of all dementia cases in memory disorders clinics [5]. Once dementia is clinically apparent, the pathological changes are irreversible; hence, it is critical that, for therapies to be affective, the underlying dementia must be accurately diagnosed at an early stage. Cognitive tests, including the mini-mental state examination (MMSE) [6], provide an assessment of overall cognitive functioning but cannot discriminate between the different dementias.

The molecular medical imaging technique of positron emission tomography (PET) and anatomical imaging from magnetic resonance (MR) imaging are able to detect focal hypometabolism (PET) and atrophy (MR), which are characteristics of neurodegenerative disorders [7–10]. These imaging techniques are dependent upon the skill and experience of the reader and interpretation can be time-consuming and prone to operator bias. A computer-aided automated dementia classification, thus, would provide a useful “second opinion.” Automated dementia classification, however, is a challenging task, especially early in the course of the illness [11]. A feasible automated approach would be based on learning the statistical models of each dementia from a set of training samples, where each training sample is associated with a class label. Pattern detection techniques used in such a system then shift from visual inspection by an expert to image-based feature extraction and selection. Patterns that have been considered include global features, computed from the entire brain volume, and local features such as statistics, histograms, and gradients calculated from volumes of interest (ROIs) [12–14]. Advanced pattern classification techniques, including the K-means clustering [15], artificial neural network [9, 10], and support vector machine (SVM) [11, 12], have also been applied. Davatzikos et al. [16] used the multiscaled principal component analysis (PCA) to extract image features in T1-weighted MR images and applied those features to a nonlinear SVM to generate a real-valued score for each MR study to differentiate AD and FTD from normal controls (NCs). Hinrichs et al. [17] separated AD from patients with mild cognitive impairment (MCI) and NCs by applying features extracted from MR and PET images to the multikernel learning (MKL) machine. Zhang et al. [18] extended this work to a grid-search process to generate the optimal kernel weights for the MKL machine and improved the dementia classification.

Our aim was to develop an automated approach to classify AD, FTD, and NCs using a generic computer-aided system to analyze 18-fluorodeoxyglucose (^18^F-FDG) PET images. In our previous work, we used global and local features from parametric FDG-PET images to identify the different dementias [19]. The global features were obtained by applying the entire gray matter volume to the linear transformation derived from the PCA [20]. Local features were defined as the statistics of voxel values in anatomical volumes of interests (VOIs) and we adopted the AdaBoost technique to adaptively combine those feature groups [21]. In this paper, we propose the GA-MKL algorithm, which is a hybrid feature extraction, selection, and classification approach, for the automated identification of AD, FTD, and NCs using parametric FDG-PET. Based on the observation that the volume loss and reduced glucose metabolism in FTD are mainly seen in the frontal and temporal lobes and the AD changes are usually located more posteriorly in the parietotemporal cortices, our approach extracted three groups of local features in 116 anatomical VOIs characterizing the average glucose metabolism rate in each VOI, the variation of metabolic rates in each VOI, and the asymmetry of metabolic rates in left-right VOI pairs. We selected a subset of the most effective features from each group to reduce the redundancy in these groups and formulated the feature selection task as an optimization problem and solved it using the genetic algorithm (GA), which has the ability to search the global optimum. To identify each clinical condition, we applied three groups of selected features to the MKL machine, in which the weighting parameter of each kernel was automatically estimated. We compared our GA-MKL algorithm to two state-of-the-art dementia classification methods on 129 clinical studies.

## 2. Materials and Methods

### 2.1. Data Acquisition

We used 129 clinical brain FDG PET studies with a clinical diagnosis of AD in 46 and FTD in 43 and there were 40 NCs. All studies were acquired on an ECAT 951/R whole body PET scanner (Siemens/CTI, Knoxville, TN, USA) in the Department of Molecular Imaging at the Royal Prince Alfred Hospital (Sydney, Australia) between 1998 and 2007. Approximately 400 MBq of ^18^F-FDG was infused at a constant rate over a 3-minute period. Two arterialised-venous blood samples were taken at 10 minutes and 45 minutes after injection to calibrate the population-based input function using a method published previously [22]. PET scanning commenced at least 30 minutes after tracer injection with scan duration of 20 minutes. Each FDG-PET data volume had a dimension of 128 × 128 × 31 and a voxel size of 1.84 × 1.84 × 3.38 mm^3^. The autoradiographic method [23] was used to calculate parametric images of cerebral metabolic rate of glucose consumption (CMRGlc).

### 2.2. Spatial Normalization

**Figure 1:**
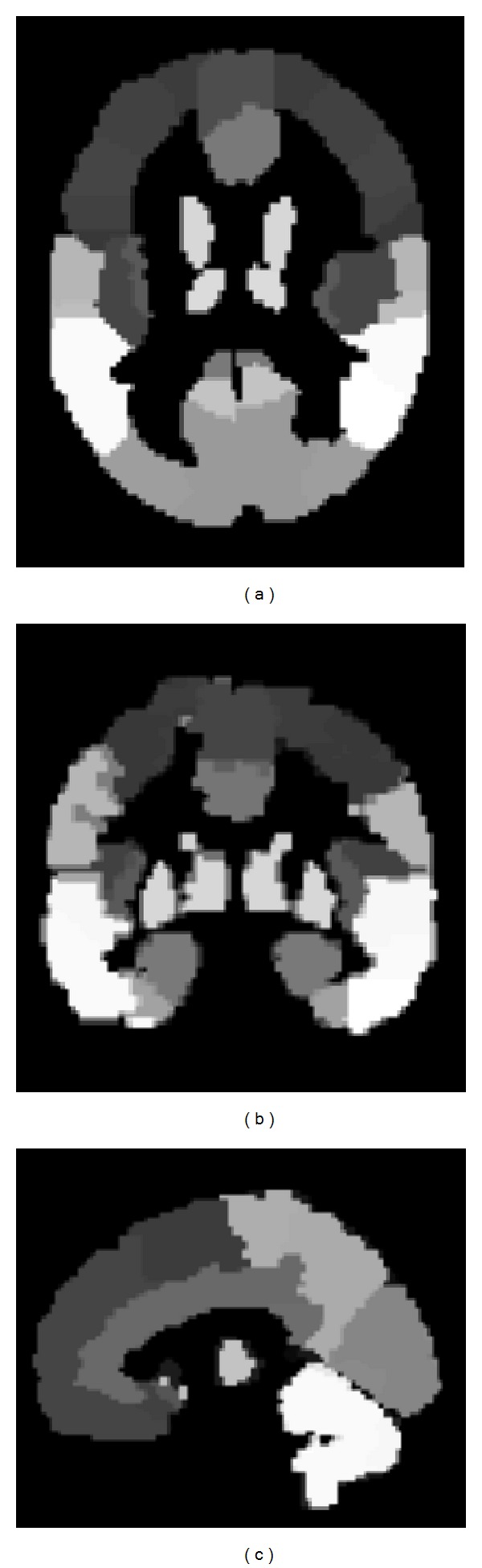
Transverse (a), coronal (b), and sagittal (c) views of the AAL cortical parcellation map.

To differentiate cortical gray matter from white matter, we used the automated anatomical labeling (AAL) cortical parcellation map [24], which was built by applying a set of anatomical parcellation rules to the spatially normalized single subject high resolution T1 volume provided by the Montreal Neurological Institute (MNI) [25]. It consisted of 116 anatomical VOIs, including 54 left-right pairs. The transverse, coronal, and sagittal views of the AAL cortical parcellation map are displayed in Figure 1. In these images, different gray levels indicate different anatomical volumes. A full list of all anatomical VOIs can be found in the AAL package [24].

The AAL cortical parcellation map is well aligned with the template brain PET image supplied with the statistical parametric mapping (SPM, Version 8) package (Wellcome Trust Centre for Neuroimaging, London, UK) [26], which conforms to the space defined by the international consortium for brain mapping (ICBM) and approximates to the space described in the atlas of Talairach and Tournoux [27]. To map the anatomical labels from the atlas onto each study, we spatially normalized each reconstructed CMRGlc image to the SPM brain PET template using the spatial normalization procedure supplied with the SPM package. Each spatially normalized CMRGlc image had a dimension of 91 × 109 × 91 and a voxel size of 2 × 2 × 2 mm^3^.

### 2.3. Feature Extraction

After the spatial normalization, each CMRGlc image and the AAL cortical parcellation map lie in the same coordinate system. Consequently, 116 anatomical VOIs can be identified on each study by using the corresponding voxel labels in the AAL cortical parcellation map. For each study *i*, we used the mean and standard deviation of voxel values in each anatomical VOI in the spatially normalized CMRGlc image as two groups of image features, denoted by *X*_*i*_^(*M*)^ = [*x*_*i*,1_^(*M*)^, *x*_*i*,2_^(*M*)^,…, *x*_*i*,116_^(*M*)^] and *X*_*i*_^(*S*)^ = [*x*_*i*,1_^(*S*)^, *x*_*i*,2_^(*S*)^,…, *x*_*i*,116_^(*S*)^]. Metabolic asymmetry is also prominent in the dementia syndromes; hence, we used the difference between the mean voxel values of each of 54 left-right VOI pairs as the third group of features, denoted by *X*_*i*_^(*A*)^ = [*x*_*i*,1_^(*A*)^, *x*_*i*,2_^(*A*)^,…, *x*_*i*,54_^(*A*)^]. As a result, for the *i*th brain PET study, we extracted three groups of image features, 286 features in total, denoted by *X*_*i*_ = {*X*_*i*_^(*M*)^, *X*_*i*_^(*S*)^, *X*_*i*_^(*A*)^}. These three groups of features characterized the average cerebral metabolic rate and the spatial variation of cerebral metabolic rates in each VOI and the asymmetry in glucose metabolism between the left and right brain hemispheres. Since the image features could have a variable dynamic range over all studies, each of the 286 features was normalized by subtracting the sample mean and dividing by the sample standard deviation before applying subsequent processing.

### 2.4. Feature Selection

Although 116 anatomical VOIs were used in feature extraction, not every VOI was equally important for dementia classification. So we removed those features that contributed little to the classification. Feature selection was performed on a group-by-group basis. Let the *C*th group of features extracted from *N* brain PET studies be denoted by *X* ^(*C*)^ = [*X*_1_^(*C*)^, *X*_2_^(*C*)^,…, *X*_*N*_*C*__^(*C*)^], where *X*_*d*_^(*C*)^ = [*x*_1,*d*_^(*C*)^,*x*_2,*d*_^(*C*)^,…,*x*_*N*,*d*_^(*C*)^]^*T*^ and *N*_*C*_ is the number of features in this group. Let Φ be a *N*_*C*_-dimensional binary vector, where 1 means that the corresponding feature is selected and 0 means that the feature is discarded. Each binary vector Φ acts as a mask to “filter” all features to preserve the selected ones. The subset of selected features can be denoted by Φ⊚*X* ^(*C*)^. Our aim was to identify a subset of features that produce the most accurate classification of all studies. Let the accuracy of classifying the data set *X* ^(*C*)^ with the classifier *c* be formally represented as *f*_*c*_(*X* ^(*C*)^); the feature selection can be formulated into the following optimization problem:

(1) $$\begin{array}{c} \Phi^{*}=\arg\underset{\Phi }{\max}f_{c}\left(\Phi ⊚X^{\left(C\right)}\right). \end{array}$$

Due to the relatively large number of features, it is not feasible to solve this combinatorial optimization by attempting every possible combination of features. Thus, we used the binary-coded GA (bGA) to find a satisfactory feature subset [28]. The GA is a heuristic-guided parallel and stochastic search strategy, searching through an evolving population of individuals. The bGA-based optimization started with a population of 500 randomly initialized binary individuals, each representing a candidate solution Φ_*i*_ and having the fitness value *f*_*c*_(Φ_*i*_⊚*X* ^(*C*)^). Since the number of studies was relatively small and the number of features is large, SVM with a linear kernel function was adopted as the classifier [29]. To efficiently use all available studies, the 10-fold cross validation scheme was performed. All PET studies were randomly partitioned into 10 equal size subsamples. Of the 10 subsamples, a single subsample was retained as the validation data for testing the classifier, and the remaining 9 subsamples were used as training data. The cross-validation process was then repeated 10 times, with each of the 10 subsamples used once as the validation data. The fitness of the individual that represented the solution Φ_*i*_ was then defined as the average classification error achieved in the 10-fold cross validation when the selected features Φ⊚*X* ^(*C*)^ were used.

During the evolutionary optimization process, each new generation was created by using several genetic operators, including the best solution inheritance, roulette wheel selection, one-point crossover, random mutation, and gene modification. Since the classifier prefers lower dimensionality of the feature space, gene modification is designed to produce new solutions by modifying the current optimal solution by discarding 1 to 3 selected features which make the least contribution to the fitness. To avoid the optimization process being trapped in a local maximum, we used a variable mutation probability, given as follows:

(2) $$\begin{array}{c} {p_{m}}^{\left(n+1\right)}=\{\begin{array}{cc} {p_{m}}^{\left(n\right)}, & {f_{m}}^{\left(n\right)}>{f_{m}}^{\left(n-1\right)},\\ \alpha_{pm}\cdot {p_{m}}^{\left(n\right)}, & \text{otherwise}, \end{array} \end{array}$$

where *α*_*pm*_ was the increasing rate of mutation probability and *p*_*m*_^(*n*)^ and *f*_*m*_^(*n*)^ are the mutation probability and the highest fitness of the *n*th generation. When the mutation probability reaches its threshold *T*_*pm*_, it will be reset to its initial value *p*_*m*_^(0)^ to prevent the bGA from degenerating to random searching. Another operator is triggered when the evolution has been halted for more than 4 generations. In this case, all individuals whose fitness equals the highest fitness will be replaced by their offspring produced by mutating. This operator aims to diversify genes in the population and thus speed up the evolution. Finally, the evolution terminates when the predetermined number of generations is reached. In the final population, let the individual with the highest fitness be denoted by Φ*. The optimal subset of features we selected is *X* ^∗(*C*)^ = Φ*⊚*X* ^(*C*)^ and the overall selected feature set is denoted by X* = {*X* ^∗(*M*)^, *X* ^∗(*S*)^, *X* ^∗(*A*)^}.

### 2.5. MKL-Based Classification

Based on the selected features, the classification of FDG-PET studies was obtained by using a MKL machine, which is a linear combination of soft-margin SVMs with multiple linear and nonlinear kernels [30, 31]. The prototype soft margin SVM is defined as the following minimization problem:

(3)  min⁡w,ξ,b (C0∑i=1NTξi+12||w||2),  s.t.    ti(w·Xi∗−b)≥1−ξi, ξi≥0,

where *ξ*_*i*_ is the slack variable for each data sample *i*, **w** is the vector orthogonal to the decision hyperplane, *t*_*i*_ is the target value for feature vector *X*_i_*, and *N*_*T*_ is the number of training cases. The dual optimization problem of (3) derived using the Lagranian technique is

(4)  max⁡α⁡ (∑i=1NTαi−12∑i,j=1NTαiαjtitjXi∗TXj∗),  s.t.    0≤αi≤C0, ∑i=1NTαiti=0,

where **α** = [*α*_1_, *α*_2_,…, *α*_*N*_*T*__] is the Lagranian multiplier. Generally, the inner product *X*_*i*_^∗*T*^ *X*_j_* can be denoted by a linear kernel function *K*(*X*_i_*, *X*_j_*). In this study, we employed the linear, second-order polynomial and Gaussian kernel functions to handle three groups of selected features. Thus, the objective function in (4) can be rewritten as

(5) $$\begin{array}{c} \underset{\alpha }{\max}\left(\sum_{i=1}^{N_{T}}\alpha_{i}-\frac{1}{2}\sum_{i,j=1}^{N_{T}}\alpha_{i}\alpha_{j}t_{i}t_{j}\sum_{C\in \left\{M,S,A\right\}}\beta_{C}K_{C}\left(X_{i}^{*(C)},X_{j}^{*(C)}\right)\right), \end{array}$$

where *β*_*C*_ > 0 is the weighting parameter for the *C*th feature group and three kernel functions are as follows:

(6) KM(Xi∗(M),Xj∗(M))=(Xi∗(M))TXj∗(M),KS(Xi∗(S),Xj∗(S))=[(Xi∗(S))TXj∗(S)+1]2,KA(Xi∗(A),Xj∗(A))  =exp⁡(−[Xi∗(A)−Xj∗(A)]T[Xi∗(A)−Xj∗(A)]2σ2).

Different from traditional MKL approaches [17, 18], the optimal weight vector [*β*_*M*_, *β*_*S*_, *β*_*A*_] in our approach was automatically estimated by the real-coded GA with the procedures similar to those used in feature selection. The Lagranian multiplier **α** was obtained by using the traditional SVM technique. For each test case *X*_j_*, we first calculated the vote for each possible class label *t*_*i*_ (*i* = 1,2,…, *N*_*T*_) using the trained MKL:

(7) $$\begin{array}{c} y_{ji}=\alpha_{i}\sum_{C\in \left\{M,S,A\right\}}\beta_{C}K_{C}\left(X_{i}^{*(C)},X_{j}^{*(C)}\right). \end{array}$$

The test case was grouped into the class that had the maximum accumulated votes.

### 2.6. Summary

**Figure 2:**
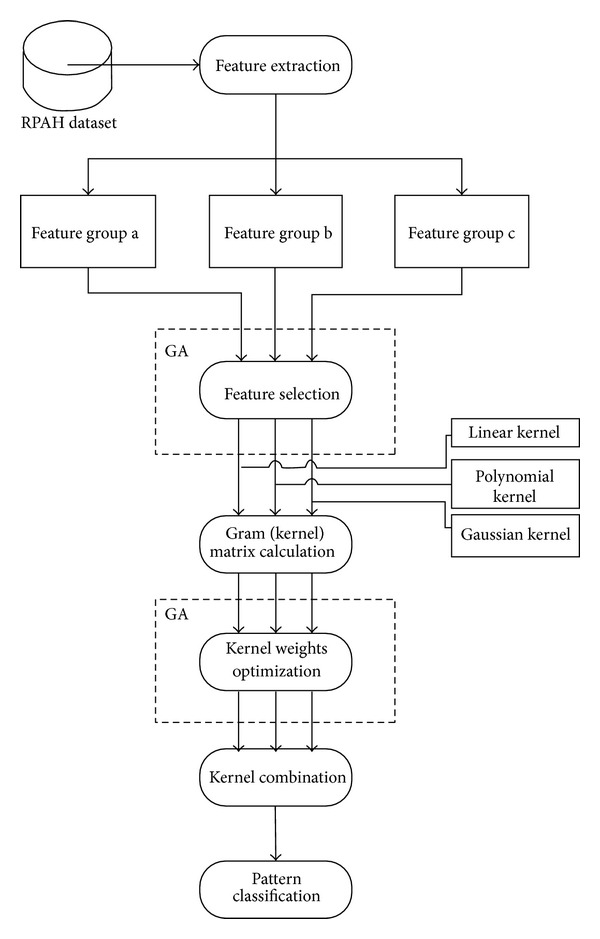
Scheme of the proposed GA-MKL dementia classification algorithm.

The scheme of the proposed GA-MKL dementia classification algorithm is illustrated in Figure 2.

### 2.7. Evaluation

We compared our GA-MKL algorithm to the methods reported by Zhang et al. [18] and Xia et al. [21], which employ the GA-based feature selection and MKL-based classification, respectively. We adopted a 10-fold cross validation scheme to ensure a comprehensive comparison. In each experiment, 90% of studies were used to train the feature selection and classification system and the other 10% of studies were left for testing. In this way, it was guaranteed that test data were used to train the algorithm. After the experiment was repeated 10 times, each study was then tested once. The performance of each approach was evaluated for overall classification accuracy, which was calculated as the percentage of correctly classified studies. Similar experiments were performed to differentiate studies from each pair, including AD versus NCs, FTD versus NCs, and AD versus FTD. For each pair, the performance of each algorithm was measured by accuracy, sensitivity (true positive rate), and specificity (true negative rate), which were defined as follows:

(2.7) $$\begin{array}{c} \text{accuracy}=\frac{\text{number}\;\text{of}\;\text{correctly}\;\text{classified}\;\text{cases}}{\text{total}\;\text{number}\;\text{of}\;\text{cases}},\\ \text{sensitivity}=\frac{\text{number}\;\text{of}\;\text{true}\;\text{positive}\;\text{cases}}{\text{number}\;\text{of}\;\text{positive}\;\text{cases}},\\ \text{specificity}=\frac{\text{number}\;\text{of}\;\text{true}\;\text{negative}\;\text{cases}}{\text{number}\;\text{of}\;\text{negative}\;\text{cases}}. \end{array}$$

## 3. Results and Discussion

Table 1 lists the accuracy of the three algorithms. It shows that the proposed GA-MKL algorithm achieves an identification accuracy of 94.62%, substantially higher than the accuracy achieved by other two algorithms.

In Table 2, the sensitivity, specificity, and accuracy for the pairs of conditions are recorded. Our GA-MKL algorithm showed the best results, in particular, when separating FTD from NCs. However, in the differentiation of AD from FTD, our algorithm had slightly lower specificity when compared to Zhang et al. [18], but it still produced the highest sensitivity and accuracy across the three algorithms.

All algorithms have difficulty in separating AD from FTD and this problem is also recognized in clinical practice [32]. We applied the paired *t*-test to these image features to explicitly display this problem. The percentage of data in each group rejecting the hypothesis that those data are drawn from the normal distribution with an identical mean and variance is shown in Table 3. We found that the majority of data rejected the identical distribution hypothesis when mixing dementia cases with normal controls, whereas only 43.7% data rejected this hypothesis when AD and FTD cases were grouped together. These results show that the features extracted and selected by our algorithm are more capable of separating AD or FTD cases from normal controls but less capable of differentiating AD from FTD. Image features that can more effectively characterize the asymmetric hypometabolism in FTD will be further investigated in our future work.

In our GA-MKL algorithm, image features were extracted in all anatomical VOIs defined by the AAL cortical parcellation map [24], without evaluating the relevance between each VOI and the dementia type. Hence, feature selection plays a pivotal role in improving the performance of dementia classification. We formulated this task as a maximization problem in (1) and solved it using the GA. The optimal subset of features can be determined in alternative ways. For example, as a classical statistical hypothesis test method, *t*-test has been adopted to select features in the form of bagged *t*-test [33] and modified *τ*-statistics [34] in research areas such as genotype data classification [35] and hippocampal shape features discrimination [36]. In our work, we also attempted to select features based on the *t*-test in an iterative manner such as the forward/backward search [37].  Table 4 gives the classification accuracy when features are not selected or selected by using the *t*-test method or the proposed GA. Our results show that, if all features are used or the features selected by *t*-test, our algorithm has much lower accuracy. This is mainly because the features with less discriminatory power do not contribute to the classification and decrease the performance of a classifier, since they increase the dimensionality of the feature space. It also explains why dimensionality reduction always plays a pivotal role in high-dimensional pattern classification. In the meantime, the *t*-test-based feature selection method ignores the interrelationship among multiple features. Nevertheless, it is widely recognized that a feature considered to be useless in itself may help improve the overall separability of the sample data if it is combined with other features [38].

We chose the MKL machine to classify the FDG-PET studies. Lanckriet et al. [31] reported that MKL is a semidefinite programming mechanism, which is bonded to SVM naturally and is suitable for handling data from heterogeneous data sources. The selection of the kernel function for each feature group is also critical to our algorithm. In our experiments, 62 out of 116 features were selected in the first group, 58 out of 116 in the second group, and 23 out of 54 in the third group. The advantage of SVM is that low-dimensional features can be converted into high-dimensional space, in which data samples may have improved separability, by using nonlinear kernel functions. Thus, we applied the linear and second-order polynomial kernel functions to the first and second groups of features, which have higher dimensionality, and applied the nonlinear Gaussian kernel to the third group of features. We swapped the three kernel functions and the accuracy is listed in Table 5. It shows that the settings that we used achieved the best results.

It should be noted that we used the clinical diagnosis as gold standard classification for each PET study. It is accepted that the clinical diagnosis can be problematic and a definitive diagnosis can only be made with pathological confirmation after death. The patients in our cohort were all assessed by experienced neurologists/geriatricians in a dementia clinic setting and cases without a diagnosis of probable AD or FTD were excluded. Hence, a relatively accurate classification rate was achieved by all algorithms. However, it would be expected that all the algorithms would perform poorly if atypical studies were included in training and testing. So we also selected 12 AD, 11 FTD, and 11 normal cases, which were described by doctors as “atypical,” and added them to the dataset. The performance of three algorithms on these 163 studies is shown in Table 6. All algorithms have poorer accuracy but our GA-MKL algorithm was still the most accurate.

## 4. Conclusion

We have proposed a novel dementia classification algorithm, namely, the GA-MKL algorithm, which extracts three groups of features, selects a subset of features from each group using the GA, and classifies the selected features using the MKL machine with automatically estimated weighting parameters. Our results show that the GA-MKL algorithm produces improved sensitivity, specificity, and accuracy when compared to two other state-of-the-art approaches. We chose FDG-PET images to test our algorithm, but we suggest that our algorithm is generic and can be applied to other scanning techniques such as amyloid imaging scans using the Pittsburgh compound B (PiB) [39]. In future work, we will apply our methodology to these newer PET ligands.

## Figures and Tables

**Table 1:** Accuracy of three dementia identification algorithms on 129 FDG-PET studies.

	Algorithm in [18]	Algorithm in [21]	Proposed GA-MKL algorithm
Accuracy	89.23%	91.47%	**94.62%**

The bold font refers to the best performance obtained in each test.

**Table 2:** Performance of the algorithm in binary comparisons.

	Algorithm in [18]	Algorithm in [21]	Purposed GA-MKL algorithm
AD versus normal (86 studies)			
Sensitivity	93.48%	91.30%	**97.82%**
Specificity	97.50%	97.50%	**100%**
Accuracy	91.81%	93.19%	**98.89%**

FTD versus normal (83 studies)			
Sensitivity	93.02%	95.35%	**100%**
Specificity	97.50%	95.00%	**100%**
Accuracy	97.64%	95.42%	**100%**

AD versus FTD (89 studies)			
Sensitivity	91.30%	91.11%	**95.65%**
Specificity	**97.67%**	85.71%	95.35%
Accuracy	90.83%	87.36%	**94.55%**

The bold font refers to the best performance obtained in each test.

**Table 3:** Percentage of data in each group rejecting the hypothesis in paired *t*-test.

	AD versus normal (86 studies)	FTD versus normal (83 studies)	AD versus FTD (89 studies)
% of rejecting the hypothesis	81.80%	71.70%	43.70%

**Table 4:** Accuracy of our algorithm with different feature selection methods.

	Without feature selection	*t*-test-based feature selection	Proposed feature selection
Accuracy	80.03%	83.96 %	**94.62%**

The bold font refers to the best performance obtained in each test.

**Table 5:** Accuracy of our algorithms when different kernel functions were used.

Trials	1st feature group *X*_*i*_^(*M*)^	2nd feature group *X*_*i*_^(*S*)^	3rd feature group *X*_*i*_^(*A*)^	Accuracy
1	Linear kernel	Polynomial kernel	Gaussian kernel	**94.62%**
2	Linear kernel	Gaussian kernel	Polynomial kernel	93.60%
3	Polynomial kernel	Linear kernel	Gaussian kernel	93.05%
4	Polynomial kernel	Gaussian kernel	Linear kernel	90.15%
5	Gaussian kernel	Linear kernel	Polynomial kernel	90.20%
6	Gaussian kernel	Polynomial kernel	Linear kernel	93.16%

The bold font refers to the best performance obtained in each test.

**Table 6:** Accuracy of the three algorithms on the larger dataset (*n* = 163).

	Algorithm in [18]	Algorithm in [21]	Purposed GA-MKL algorithm
Accuracy	71.07%	82.83%	**89.99%**

The bold font refers to the best performance obtained in each test.
